# Targeted sequencing of genes associated with the mismatch repair pathway in patients with endometrial cancer

**DOI:** 10.1371/journal.pone.0235613

**Published:** 2020-07-07

**Authors:** Ashish Kumar Singh, Bente Talseth-Palmer, Mary McPhillips, Liss Anne Solberg Lavik, Alexandre Xavier, Finn Drabløs, Wenche Sjursen

**Affiliations:** 1 Department of Medical Genetics, St. Olavs Hospital, Trondheim, Norway; 2 Department of Clinical and Molecular Medicine, Faculty of Medicine and Health Sciences, NTNU—Norwegian University of Science and Technology, Trondheim, Norway; 3 School of Biomedical Science and Pharmacy, Faculty of Health and Medicine, University of Newcastle and Hunter Medical Research Institute, Newcastle, Australia; 4 Department of Research and Development, Møre og Romsdal Hospital Trust, Molde, Norway; 5 NSW Health Pathology, Molecular Medicine, John Hunter Hospital, Newcastle, NSW, Australia; Leiden University Medical Centre, NETHERLANDS

## Abstract

Germline variants inactivating the mismatch repair (MMR) genes *MLH1*, *MSH2*, *MSH6* and *PMS2* cause Lynch syndrome that implies an increased cancer risk, where colon and endometrial cancer are the most frequent. Identification of these pathogenic variants is important to identify endometrial cancer patients with inherited increased risk of new cancers, in order to offer them lifesaving surveillance. However, several other genes are also part of the MMR pathway. It is therefore relevant to search for variants in additional genes that may be associated with cancer risk by including all known genes involved in the MMR pathway. Next-generation sequencing was used to screen 22 genes involved in the MMR pathway in constitutional DNA extracted from full blood from 199 unselected endometrial cancer patients. Bioinformatic pipelines were developed for identification and functional annotation of variants, using several different software tools and custom programs. This facilitated identification of 22 exonic, 4 UTR and 9 intronic variants that could be classified according to pathogenicity. This study has identified several germline variants in genes of the MMR pathway that potentially may be associated with an increased risk for cancer, in particular endometrial cancer, and therefore are relevant for further investigation. We have also developed bioinformatics strategies to analyse targeted sequencing data, including low quality data and genomic regions outside of the protein coding exons of the relevant genes.

## Introduction

Cancer is a life-threatening disease, with 18.1 million new cancer cases and 9.6 million cancer deaths worldwide in 2018 [1]. There is an increasing number of cases every year, and it has become an enormous burden to society. With longer life span, increased population and changed lifestyle, we can expect to have even more cases of cancer in the future. Among many types of cancers, incidences of endometrial cancer (EC) have increased worldwide in recent years [2], and it is currently the most common gynecological disease in western world [3]. This is the sixth most commonly diagnosed cancer and the fourteenth leading cause of death for women worldwide, with 380,000 estimated new cases in 2018 [1]. In Europe around 88,000 women get affected with EC every year, making EC the fourth most common cancer in women and tenth most common cancer among cancer related deaths [4]. With these high rates, it is important to diagnose EC at early and treatable stages. Environmental factors, changed lifestyle, high BMI, hypertension, menstrual irregularities and hormonal imbalances can play important roles towards carcinogenesis [5].

Hereditary factors also contribute towards EC. Higher incidences of EC are common among close relatives of EC patients [6]. Micro-satellite instability (MSI), due to dysfunction of the DNA mismatch repair (MMR) pathway, has frequently been reported as an oncogenic mechanism in EC [7]. The MMR system corrects replication errors, in particular single nucleotide variants and insertion-deletion (INDEL) loops, and failure in this system can result in MSI. Around ~30% of EC patients have been found with hyper-mutable phenotype and MSI [7–9] induced by dysfunctional MMR. MMR dysfunction is the cause of Lynch syndrome (LS), an autosomal dominant inherited cancer susceptibility syndrome, also known as hereditary non-polyposis colon cancer (HNPCC). LS is characterized by early-onset epithelial cancers. Individuals affected with LS have high risk of colorectal cancer (CRC) and EC, in addition to an increased risk of other epithelial malignancies like bowel, stomach, ovary, bladder, or pancreas cancer to mention a few [10]. Life-time risk of LS-affected individuals for EC is 33–61% and for CRC 40–80% [11, 12]. Not all CRC and EC with MMR deficiency are due to germline mutation, rather, most of the cases are sporadic cancers occurring due to epigenetic silencing of the MMR gene *MLH1* by DNA methylation [13–15]. It is important to identify EC cases with LS as they require regular surveillance, like colonoscopy. Given the high risk for developing new primary cancers, including CRC, this has been proven to reduce the overall mortality of the disease. If mutations in MMR genes are identified it will give the patient a diagnosis of LS and also enable at-risk relatives to be informed about their cancer risks. In addition, if pathogenic variants are identified in novel genes it could possibly explain why pathogenic variants are identified only in approximately 50% of families with a clinical diagnosis of LS (i.e. they fulfil the Amsterdam criteria) [16].

Since the rate of MSI tumours reported in EC cases is higher (30%) compared to other cancers (ie 15% in CRC), illustrating that an abnormal DNA MMR pathway plays a role in EC tumorigenesis, we decided to look into a more extended set of genes than those known to be involved in LS (*MLH1*, *MSH2*, *MSH6*, *PMS2* and deletions in *EPCAM1*). In the present study, 22 genes (both coding and noncoding parts) involved in the MMR pathway were sequenced in DNA from 199 sporadic EC patients. Targeted next generation sequencing (NGS) was used, aiming to identify novel genetic variation like substitutions, insertions/deletions (indels) and structural alterations (e.g. copy number variations) that may lead to the multi-step process of carcinogenesis.

## Materials and methods

The study was performed on DNA extracted from full blood from 199 patient samples from a study which included consecutively recruited women with histologically confirmed EC (sporadic cases) who presented for treatment at the Hunter Centre for Gynaecological Cancer, John Hunter Hospital, Newcastle, New South Wales, Australia between the years 1992 and 2005 [17]. Blood samples were taken in year 2005 for the present study. The study has been approved by Hunter New England (HNE) Human Research Ethics Committee (HNE HREC: 05/03/09/3.14). Written informed consent was obtained from all participants.

### Targeted next generation sequencing (NGS)

Targeted NGS sequencing was performed on the 199 patient samples, using an Illumina MiSeq [18] instrument. Initially 12 runs were performed to sequence the samples; later 15 samples were re-sequenced due to low quality of the initial sequencing. The target regions (all introns, exons, 5’ and 3’ UTRs) of 22 MMR genes (*MLH1*, *MSH2*, *MSH6*, *PMS2*, *MSH3*, *PMS1*, *MLH3*, *EXO1*, *RFC1*, *RFC2*, *RFC3*, *RFC4*, *RFC5*, *PCNA*, *LIG1*, *RPA1*, *RPA2*, *RPA3*, *POLD1*, *POLD2*, *POLD3* and *POLD4*) with a total size of 1.213 Mb were captured using 6961 probes and the Illumina Nextera Rapid Capture Enrichment Kit (custom, 96 samples). An overview of these 22 genes, their function and associated phenotypes are shown in Table 1. Sequencing was performed at the Medical Genetics Laboratory at Hunter Medical Research Institute (HMRI), University of Newcastle, Australia.

**Table 1:** List of genes in target panel.

Gene		Gene function (Info source: NCBI-gene)	Phenotype (Info source: OMIM)	OMIM ID
MLH	*MLH1*	Encodes a protein which heterodimerizes with MMR endonuclease PMS2 to form MutL alpha.	Colorectal cancer, hereditary nonpolyposis, type 2; Muir-Torre syndrome; Mismatch repair cancer syndrome	120436
	*MLH3*	Member of the MutL-homolog (MLH) family, maintains genomic integrity during DNA replication and after meiotic recombination.	Colorectal cancer, hereditary nonpolyposis, type 7; Colorectal cancer, somatic; Susceptibility to Endometrial cancer	604395
MSH	*MSH2*	Forms 2 different heterodimers: MutS alpha (MSH2-MSH6 heterodimer) and MutS beta (MSH2-MSH3 heterodimer) which binds to DNA mismatches to initiate DNA repair	Colorectal cancer, hereditary nonpolyposis, type 1; Muir-Torre syndrome; Mismatch repair cancer syndrome	609309
	*MSH3*	Forms a hetero-dimer with MSH2 to form MutS beta which forms a complex with MutL alpha heterodimer and initiates mismatch repair by binding to a mismatch.	Endometrial carcinoma, somatic; Familial adenomatous polyposis 4	600887
	*MSH6*	A component of the post-replicative DNA MMR system. Heterodimerizes with MSH2 to form MutS alpha, which binds to DNA mismatches to initiate DNA repair.	Colorectal cancer, hereditary nonpolyposis, type 5; Endometrial cancer, familial; Mismatch repair cancer syndrome	600678
PMS	*PMS1*	Forms heterodimers with MLH1. Encoded protein belongs to the DNA MMR mutL/hexB family.	Hereditary nonpolyposis colorectal cancer type 3 (HNPCC3); Lynch syndrome	600258
	*PMS2*	Forms MutL-alpha heterodimer (MLH1-PMS2 hetetrodimer) which activates endonucleolytic activity following recognition of mismatches and insertion/deletion loops by the MutS-alpha and MutS-beta heterodimers.	Colorectal cancer, hereditary nonpolyposis, type 4; Mismatch repair cancer syndrome	600259
	*EXO1*	Encodes a protein with 5' to 3' exonuclease and RNase H activities. Similar to the Saccharomyces cerevisiae protein Exo1 which interacts with Msh2 for MMR.		606063
RFC	*RFC1*	Encodes large subunit of replication factor C, a 5 subunit DNA polymerase accessory protein (DNA-dependent ATPase required for eukaryotic DNA replication and repair).		102579
	*RFC2*	Encodes 40-kD subunit, responsible for binding ATP and may help promote cell survival	Disruption of this gene is associated with Williams syndrome	600404
	*RFC3*	Encodes 38-kD subunit, responsible for binding ATP and may help promote cell survival		600405
	*RFC4*	Encodes 37-kD subunit, responsible for binding ATP and may help promote cell survival		102577
	*RFC5*	Encodes 36.5-kD subunit, responsible for binding ATP and may help promote cell survival		600407
	*PCNA*	Encodes a protein which acts as a homotrimer and helps increase the process of leading strand synthesis during DNA replication, also involved in the RAD6-dependent DNA repair pathway	ataxia-telangiectasia-like disorder-2 (ATLD2)	176740
	*LIG1*	Encodes a member of the ATP-dependent DNA ligase protein family, which functions in DNA replication, recombination, and the base excision repair process.	Mutations in gene leads to ligase-I deficiency resulting in immunodeficiency and increased sensitivity to DNA-damaging agents associated with variety of cancers	126391
RPA	*RPA1*	Encodes the subunit of heterotrimeric Replication Protein A (RPA) complex, which binds to single-stranded DNA, forming a nucleoprotein complex. Complex is involved in DNA metabolism, replication, repair, recombination, telomere maintenance.	knockdown of RPA1 in HeLa cells caused accumulation of cells in S and G2/M phases, followed by cell death	179835
	*RPA2*	Same as above		179836
	*RPA3*	Same as above		179837
POLD	*POLD1*	Encodes a catalytic subunit of DNA polymerase delta, which possesses both polymerase and 3' to 5' exonuclease activity, important for DNA replication and repair.	Colorectal cancer, Susceptibility to, CRC-10; CRCS10; Mandibular hypoplasia, deafness, progeroid features, and lipodystrophy syndrome	174761
	*POLD2*	Encodes 50-kDa catalytic subunit of DNA polymerase delta which possesses both polymerase and 3' to 5' exonuclease activity and plays a critical role in DNA replication and repair.	Expression of this gene may be a marker for ovarian carcinomas	600815
	*POLD3*	Encodes the 66-kDa subunit of DNA polymerase delta.		611415
	*POLD4*	Encodes the smallest subunit of DNA polymerase delta POLDS-P12.		611525

### Bioinformatic analysis

Raw reads (.fastq files) generated by the sequencer were processed by the following three major steps:

1. *Data pre-processing*: Raw reads were aligned to the reference genome (version hg19), and sequence alignment maps were generated. These alignment maps were used for read visualization and to call variants.
2. *Variants discovery*: The alignment maps generated from previous steps were compared against the reference genome to generate a list of nucleotide variants.
3. *Variants annotation*: Variants were annotated using different databases and tools.

**Fig 1:**
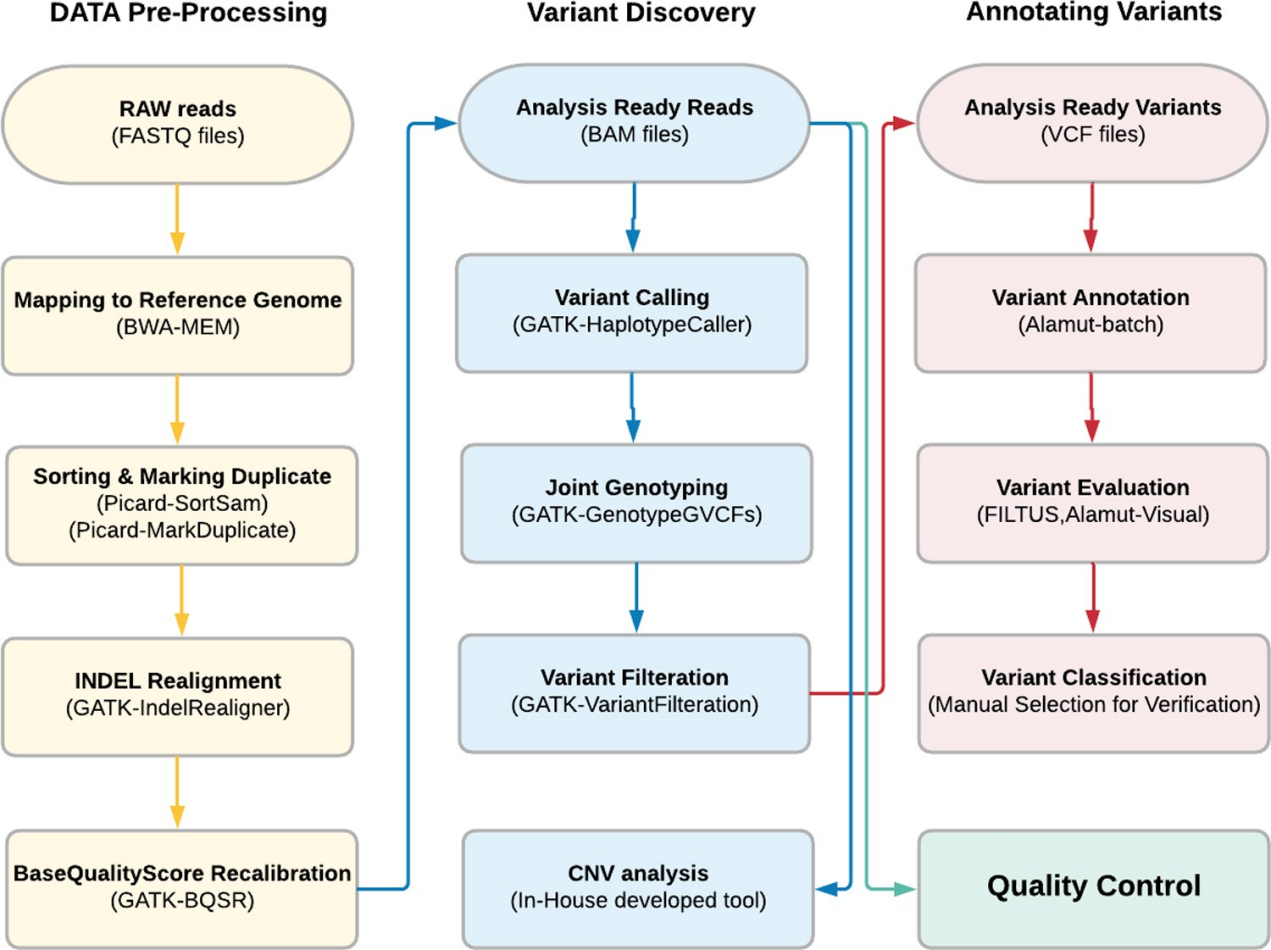
Schematic overview of the bioinformatics pipeline.

A pipeline was constructed to perform the above-mentioned steps of analysis. Detailed overview of pipeline and tools used can be found as S1 File. Schematic overview of the pipeline is shown in Fig 1.

### Filtration of variants

**Fig 2:**
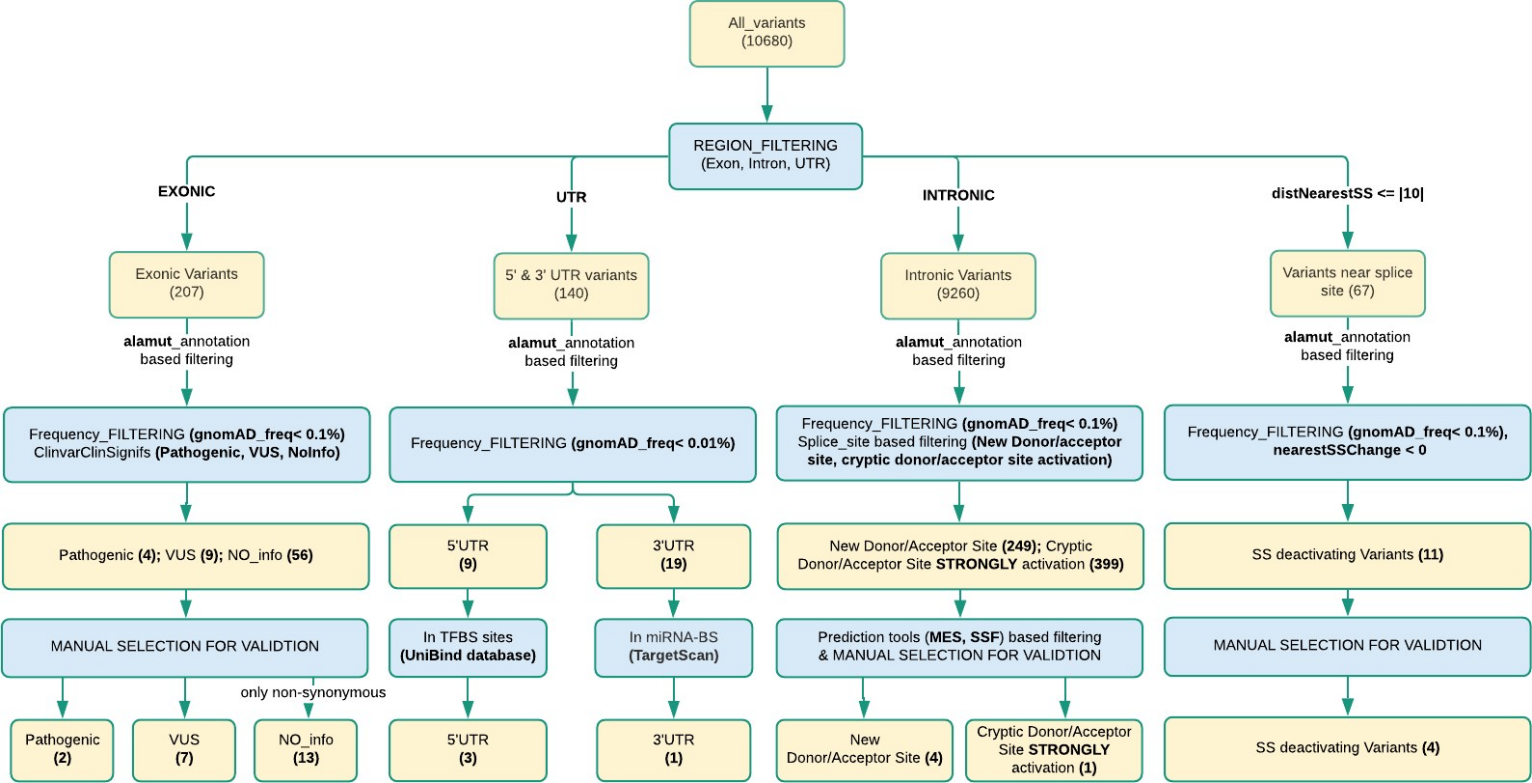
Filtering workflow and number of genetic variants detected.

All called variants were annotated by using Alamut-batch [19] before filtering. Filtus [20] was used for filtering variants. All variants were classified into 4 region-wise categories; exons, UTRs, introns, and splice sites (variant distance ≤ 10 nucleotides from nearest splice site). In the first stage of filtering, variants from all these four regions were filtered based on frequencies of variants in the gnomAD database [21]. Exonic variants, intronic variants, and variants near splice sites were filtered-in for frequencies less than 0.1% (or no frequency). UTR variants were filtered-in for frequencies less than 0.01% (or no frequency). In further stages of filtering, different strategies were adopted for every region. See Fig 2 for the workflow. Detailed filtering steps can be found in S2 File.

### Validation of variants

Sanger sequencing was performed for validation of selected variants. The fragments were amplified using AmpliTaq Gold® 360 MasterMix and 360 GC Enhancer (Life Technologies). Cycle sequencing reaction was performed with BigDye® Terminator v3.1 (Life Technologies) and subsequent capillary electrophoresis was performed on the ABI 3130xl or ABI 3730 (Life Technologies). List of primer sequences can be provided upon request. Sanger sequencing data was analysed using SeqScape Software v3.0 (Life Technologies). Some variants have not been verified by Sanger sequencing, partly due to unavailability of primers for some of these gene, but also due to logistic issues. But variants were thoroughly inspected in BAM files to assure they were likely to be true positive variants (enough coverage and an allele fraction of about 50%, between 30 and 75%).

### Interpretation and classification of DNA variants

The remaining variants after filtering were classified into 5 classes according to the American College of Medical genetics (ACMG) guidelines [22]. To determine whether these variants had been detected before, literature and databases including LOVD/InSIGHT (https://www.insight-group.org/variants/databases/) and ClinVar (https://www.ncbi.nlm.nih.gov/clinvar/) were searched. Potential pathogenicity of missense variants was interpreted using Alamut batch (annotation) [19] and Alamut Visual (interpretation) [23].

## Results and discussion

From all 199 samples, on average 99.8% of reads (per run) could be aligned to the reference genome (hg19) using BWA for alignment (see S1 File). Coverage depth of reads for samples and mean coverage depth for runs varied a lot among the 12 runs. Only 23 samples had a coverage of more than 100X (maximum 169X), and 50 samples had coverage of less than 30X (minimum 1X) (see S1 Table). Despite having multiple samples with low quality, the strategy for variant calling was uniformly applied to all samples. This was done to investigate the potential for identifying true variants even from target regions with low coverage depth. However, these low-quality data were not suitable for identification of copy number variants, and therefore CNV calling was not included in the final analysis.

**Fig 3:**
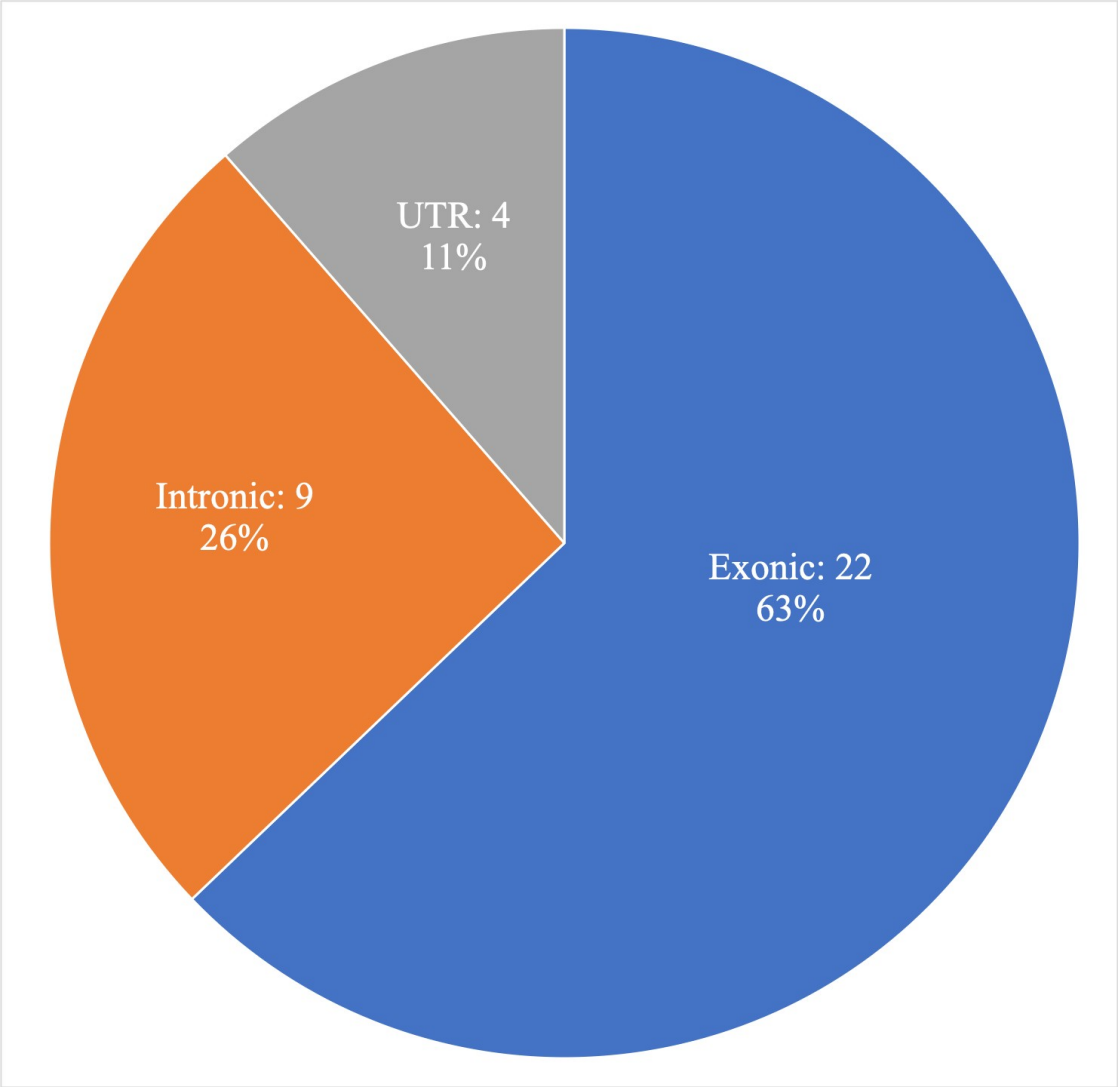
Investigated variants in different genomic regions.

In total 10,680 unique variants (substitutions and INDELs) were called using the GATK toolkit. These variants could be classified into four categories according to genomic region; exonic, intronic, UTRs and splice-site neighbourhood (≤ |10|bp). See Fig 2 for the workflow. After filtering and annotation, **22** exonic, **9** intronic (4 variants in splice-site neighbourhood) and **4** UTR variants (Fig 3) were selected for further investigation for pathogenicity as potential cancer risk variants, and these variants are described below. See Table 2 for an aggregate list. Sanger verification was performed for 21 of these 35 variants. Remaining 14 variants are not Sanger validated. These 14 variants were designated as true variants by observing BAM files.

**Table 2:** Aggregate list of variants and their classification according to the ACMG system.

Sample ID	Other Cancers^c^	Family history of Cancer^d^	Gene^a^	gNomen, cNomen, pNomen, rsID	Variant allele fraction	ClinVar / gnomAD	Class	Comments^b^	References
051783	BC	2nd° BC	*MLH1*	Chr3:g.37042548A>G, NM_000249.3:c.306+4A>G, p.(?), rs267607733	0.35	6 x VUS /0.0012%	Class 3	Activation of a cryptic donor site and the skipping of exon 3 in an ex vivo splicing minigene assay	[24]
060337	BC	NO	*MLH1*	Chr3:g.37090414A>G, NM_000249.3:c.2009A>G, p.(Lys670Arg), rs905983196	0.49	3 x VUS / NIL	Class 3	SS: Helix	
051456	NO	NO	MSH6*	Chr2:g.48010297:G>T, NM_000179.2:c.-76G>T, p.(?)	0.64	NIL/ NIL	Class 3	No frequency, highly conserved	
051026	SC	NO	*MSH6*	Chr2:g.48018140A>G, NM_000179.2:c.335A>G, p.(Asn112Ser), rs587779934	0.44	6 X VUS /0.0025%	Class 3	New acceptor site predicted SS: Turn	
051408	NO	2nd° CRC	*MSH6*	Chr2:g.48025743C>A, NM_000179.2:c.628-7C>A, p.(?), rs373129248	0.41	6 x VUS /0.0093%	Class 3		
051476	NO	1st° EC	*MSH6*	Chr2:g.48026531C>G, NM_000179.2:c.1409C>G, p.(Ser470*)	0.48	NIL/ NIL	**Class 5**	SS: Helix	
051791	NO	1st° EC	*MSH6*	Chr2:g.48027325C>A, NM_000179.2:c.2203C>A, p.(Leu735Ile), rs786204071	0.4	6 X VUS / NIL	Class 3		
051280	NO	NO	*MSH6*	Chr2:g.48032048G>T, NM_000179.2:c.3439-1G>T, p.(?), rs587779263	0.5	8 X Pathogenic / NIL	**Class 5**		
060162	NO	2nd° OC	*MSH6*	Chr2:g.48033587_48033614dup, NM_000179.2:c.3802-4_3825dup, p.(Glu1276*)	0.46	NIL/ NIL	**Class 5**		[25]
051233	NO	1st° BC	*MSH2*	Chr2:g.47630427A>C, NM_000251.2:c.97A>C, p.(Thr33Pro), rs63751107	0.75	6 X VUS / 0.0056%	Class 3	SS: Beta strand	[26, 27, 28–35, 36]
			MSH3*	Chr5:g.79968115C>T, NM_002439.4:c.845C>T, p.(Thr282Ile), rs202184623	0.67	NIL// 0.0053%	Class 3	SS: Beta strand	
051872	3 Melanomas	NO	*MSH2*	Chr2:g.47657032G>T, NM_000251.2:c.1228G>T, p.(Gly410Cys), rs587782242	0.47	1 X VUS/ NIL	Class 3	SS: Helix	
051107	Melanoma	NO	*MSH2*	Chr2:g.47672680C>A, NM_000251.2:c.1277-7C>A, p.(?), rs375437307	0.57	3 X VUS/ 0.0037%	Class 3		
051271	BC	1st° BC & PC, 2nd° CRC	*MSH2*	Chr2:g.47710015T>G, NM_000251.2:c.2732T>G, p.(Leu911Arg), rs41295182	1	1 X VUS gnomAD: 0.0062%	Class 3	SS: Helix	[34, 37–39]
051179	SKIN SPOTS	1st° CRC	*PMS2*	Chr7:g.6045549C>A, NM_001322014.1:c.137G>T, p.(Ser46Ile), rs121434629	0.44	12 X VUS:/ 0.0169%	**Class 4**	Associated with diagnosis of CMMRD syndrome, SS: Helix	[40–47]
051300	NO	1st° CRC & PC			0.47				
051657	NO	1st° OC	*MSH3*	Chr5:g.80021325A>G, NM_002439.4:c.1394A>G, p.(Tyr465Cys), rs35009542	0.58	NIL/ 0.0202%	Class 3	SS: Helix	
051172	NO	NO	MSH3*	Chr5:g.79974804G>A, NM_002439.4:c.1232G>A, p.(Arg411His), rs764885728	0.49	NIL/ 0.0012%	Class 3		
051469	PCOS	2nd° UC	MSH3*	Chr5:g.80021327A>G, NM_002439.4:c.1396A>G, p.(Ser466Gly), rs766948921	0.51	NIL/ 0.0025%	Class 3	SS: Helix	
060161	NO	NO	MSH3*	Chr5:g.80063896C>T, NM_002439.4:c.2041C>T, p.(Pro681Ser), rs115198722	0.48	NIL/ 0.0787%	Class 3	SS: Helix	
051330	BC, SpC, LC, KC AND LiC	NO			0.55				
051610	BrT	NO	*POLD1*	Chr19:g.50905980G>A, NM_001308632.1:c.952G>A, p.(Glu318Lys), rs775232133	0.63	1 X VUS / NIL	Class 3	highly conserved DI**E**	
051406	BC	1st° BC	*RFC1*	Chr4:g.39290383A>T, NM_001204747.1:c.3445T>A, p.(*1149Argext*15), rs149767968	0.59	NIL/ 0.0065%	Class 3	Altered stop codon, extension of protein with 15 aa	
			*RPA3*	Chr7:g.7676702A>G, NM_002947.4:c.295T>C, p.(Tyr99His)	0.62	NIL/ 0.0004%	Class 3	SS: Helix	
051663	NO	1st° BC	RFC1*	Chr4:g.39306530C>A, NM_001204747.1:c.2017G>T, p.(Val673Leu), rs28903096	0.33	NIL/ 0.057%	Class 3		
051400	NO	1st° Unknown CANCER	RFC1*	Chr4:g.39346049A>CNM_001204747.1:c.208+972T>G, p.(?)	0.63	NIL/ NIL	Class 3		
051471	NO	NO	*RFC3*	Chr13:g.34392210:A>G, NM_002915.3:c.-106A>G, p.(?), rs554574193	0.57	NIL/ 0.0064%	Class 3	highly conserved	
051640	NO	2nd° CRC	*RFC4*	Chr3:g.186524157:G>A, NM_002916.3:c.-90C>T, p.(?)	0.52	NIL/ NIL	Class 3	**not** conserved	
051439	NO	NO	RFC4*	Chr3:g.186518351T>C,NM_002916.3:c.210+555A>G, p.(?), rs781729102	0.55	NIL/ 0.0387%	Class 3		
051802	UC	1st° EC	*LIG1*	Chr19:g.48640874G>A, NM_000234.2:c.1159C>T, p.(Arg387Cys), rs749929415	0.48	NIL/ 0.0018%	Class 3	SS: Beta strand	
051133	BC & OC	1st° BC	LIG1*	Chr19:g.48653350A>C, NM_002439.4:c.692T>G, p.(Phe231Cys), rs767343361	0.37	NIL/ 0.0079%	Class 3	SS: Turn	
051166	BC & OC	1st° BC	*MLH1*	Chr3:g.37048554G>A, NM_000249.3:c.453G>A, p.(Thr151 =), rs369521379	0.51	9 x VUS /0.0011%	Class 3	Last nucleotide of exon 5. ClinVar Miner: damage the nearby splice donor site (at -1 distance) and cause abnormal splicing. SS: Beta strand	
			EXO1*	Chr1:g.242020650G>T, NM_006027.4:c.409G>T, p.(Ala137Ser), rs147663824	0.55	NIL/ 0.0094%	Class 3	SS: Helix	[48]
			RPA3*	Chr7:g.7753847G>T, NM_002947.4:c.-1028+959C>A, p.(?)	0.51	NIL/ NIL	Class 3		
051267	NO	1st° PCOS	EXO1*	Chr1:g.242052986T>G, NM_130398.3:c.*84T>G, p.(?)	0.5	NIL/ NIL	Class3	Can affect miRNA binding, for miR-370-3p and miR-93-3p	
051007	NO	NO	RPA1*	Chr17:g.1785509A>G,NM_002945.4:c.1241+1524A>G, p.(?), rs536796524	0.43	NIL/ 0.0323%	Class 3	It is within 1000 bp of a region that may be important for chromatin folding (Insulator / CTCF / SMC3 / RAD21)	
051291	NO	NO	RPA3*	Chr7:g.7695875T>C,NM_002947.4:c.-757-15069A>G, p.(?), rs946965390	0.54	NIL/ NIL	Class 3	It is within 2500 bp of a region that may be important for chromatin folding (Insulator / CTCF / SMC3 / RAD21)	

^a^Variants with

* not yet verified by Sanger sequencing;

^b^SS: Variant lies in Secondary Structure (UniProt)

^c^Other cancers: BC: Breast Cancer, CRC: Colorectal Cancer, PCOS: Polycystic Ovary Syndrome, PC: Prostate Cancer, UC: Uterine Cancer, EC: Endometerial Cancer,OC: Ovarian Cancer, SC: Skin Cancer, SpC: Spine Cancer, LC: Lung Cancer, KC: Kidney Cancer, LiC:Liver Cancer, BrT: Brain Tumor

^d^Family history of cancer: 1st° & 2nd°: 1st & 2nd Degree relatives with cancer-type

### Exonic variants

A total of 207 variants were called in exonic regions of the target panel, over all samples. The variants were filtered by removing cases according to their frequency in gnomAD (> 0.1%) and annotation in ClinVar (benign/likely-benign) [49]. Of the 22 exonic variants (S3 File) that remained after filtering, there were 2 putative pathogenic variants, 7 variants of unknown significance (VUS) and 13 variants without any information (NO_Info) according to ClinVar (only non-synonymous variants).

Among these 22 variants there were 2 variants in *MLH1* (NM_000249.3). Both *MLH1* variants were classified as class 3 in pathogenicity, according to ACMG guidelines [22]. The variant c.453G>A p.(Thr151 =) is found in the last nucleotide of exon 5. It may alter the ligation of adjacent exons 5 and 6 and is predicted to be splice site deactivating by prediction tools (SSF [50], MES [51]) (nearest-SS-change score: -0.29). The first and the last three positions of the exon are an integral part of the 3’ and 5’splice site consensus sequences [52], the variant position is highly conserved (PhastCons score: 0.99), and predicted as pathogenic by UMD-predictor [53]. According to ClinVar it is classified as a likely pathogenic / VUS variant, with multiple submissions in ClinVar where many of them has a HNPCC/Lynch syndrome phenotype. With strong evidences for being a pathogenic variant, it is a candidate for further RNA/functional studies. The variant c.2009A>G p.(Lys670Arg) has no frequency in the gnomAD database, but has recently been reported in ClinVar (as VUS) and in other databases. This variant has been associated with a HNPCC phenotype and hereditary cancer-predisposing syndrome, according to ClinVar. The variant position is highly conserved (PhastCons: 1, phyloP: 4.6) and lies in a helix secondary structure of the protein. It has been predicted as pathogenic (UMD-prediction, MutationTaster).

There were also four exonic variants in *MSH6* (NM_000179.2). Two of the variants, c.335A>G p.(Asn112Ser) and c.2203C>A (p.Leu735Ile), have been classified as VUS by ClinVar. These two variants have previously been associated with Lynch syndrome, HNPCC and hereditary cancer-predisposing syndrome-like phenotype according to ClinVar and other databases. Both variants are at highly conserved positions and both have been predicted as damaging by prediction tools. We classified these two variants as class 3. Variants c.1409C>G p.(Ser470*) and c.3802-4_3825dup p.(Glu1276*) both code for “STOP gain” and are disease causing. None of these variants have entries in ClinVar or frequency in gnomAD. We classify these as class 5 variants.

Three exonic variants in *MSH2* was identified (NM_000251.2), c.97A>C p.(Thr33Pro), c.1228G>T p.(Gly410Cys) and c.2732T>G p.(Leu911Arg), with all three classified as VUS by ClinVar. All three have phenotypic association to Lynch syndrome/HNPCC and hereditary cancer-predisposing syndrome and have been predicted as pathogenic/disease-causing by many prediction tools (UMD-prediction, PolyPhen, SIFT and MutationTaster). All three variant positions are highly conserved (with high scores in PhastCons and phyloP). Variant c.97A>C p.(Thr33Pro) was identified from a low quality sample (coverage depth at variant position 4X and sample coverage 7X), but was verified as a true variant by Sanger sequencing. It has been scored with a high value for decreasing protein stability (SNPs3D [54] score: -1.08) and has been suggested as a cause of reduced mismatch binding/release efficiency compared to wild-type protein in previous studies by Ollila *et al*. [26, 27]. Variant c.1228G>T p.(Gly410Cys) has no frequency in gnomAD, but has been reported to ClinVar and is located in a helix secondary structure of the protein. It has a high score for structural change (Grantham-Distance: 159), but it is predicted not to alter protein stability (SNPs3D: +3.43). Variant c.2732T>G p.(Leu911Arg) was also identified in a low quality sample (coverage depth at variant position 5X, sample 10X). It lies in a helix secondary structure, has high score for structural change (Grantham-Distance: 102) and for decreased protein stability (SNPs3D: -1.08). These three *MSH2* variants have been classified as class 3.

A missense exonic variant in *PMS2* was also detected (NM_001322014.), c.137G>T p.(Ser46Ile), which was found in two samples. It was classified as likely pathogenic according to ClinVar, and is reported to be a founder mutation [55]. The protein region has helix-like secondary structure (UniProt [56]), and the position is highly conserved (PhastCons [57] score:1; phyloP [58] score: 6.178). It has been classified as pathogenic by several prediction tools (UMD-predictor, PolyPhen [59], SIFT [60], and MutationTaster [61], the variant has been referred to in many previous studies, and it has been considered for strongly decreased DNA mismatch repair activity. This variant was classified as class 4.

One exonic variant in *POLD1* was identified (NM_001308632.1), c.952G>A p.(Glu318Lys), was classified as VUS according to ClinVar. It was called in a low-quality sample (coverage depth at variant position 11X, sample’s mean coverage depth 22X). The position is highly conserved (PhastCons: 1, phyloP:3.9). The variant is in the DNA binding cleft of the exonuclease active domain of *POLD1*, it has a high score for decreased protein stability (SNPs3D: -2.68), and is predicted as damaging by prediction tools. A previous study has predicted it to be disease causing [62]. However, functional studies are needed to confirm pathogenicity, and therefore it was classified as class 3.

Exonic variants were also found in five other genes; *MSH3*, *LIG1*, *RFC1*, *EXO1* and *RPA3*. All these variants were classified as class 3. In *MSH3* (NM_002439.4) variants were c.1394A>G p.(Tyr465Cys), c.845C>T p.(Thr282Ile), c.1232G>A p.(Arg411His), c.1396A>G p.(Ser466Gly), c.2041C>T p.(Pro681Ser). In *LIG1* (NM_000234.2) variants were c.1159C>T p.(Arg387Cys) and c.692T>G p.(Phe231Cys). In *RFC1* (NM_001204747.1) this was c.3445T>A p.(*1149Argext*15), which introduces a “STOP loss” and extension of 15 amino acids in the product protein and c.2017G>T p.(Val673Leu); in *EXO1* (NM_006027.4) c.409G>T p.(Ala137Ser); In *RPA3* (NM_002947.4) it was c.295T>C p.(Tyr99His).

### Intronic variants

Among all detected variants, 9,260 were identified as intronic, which was ~97% of all variants. Intronic regions of human DNA, being extraordinarily larger in comparison to other regions, it is expected to find most of the variants in these non-coding regions. After frequency-based filtering (< 0.1%), this list was reduced to 4,197 variants, which was further reduced by splice site related filtering, using strict filtering criteria to reduce the large number of variants. These variants were filtered for two categories, first for “New Donor/Acceptor site” and then for “Cryptic Donor/Acceptor Site STRONG activation” (see S2 File for filtering details). We found in total five variants, with four variants in the first category and one in the second (see S3 File). Two of these variants were in *RPA1* and *RPA3*, and have been predicted as new acceptor site, two were in *RFC1* and *RFC4* and have been predicted as new donor sites, and one was in *RPA3* and has been predicted to give strong activation of a cryptic donor site.

According to the Human Gene Mutation Database (HGMD) more than 10% of all disease-causing hereditary mutations are splice site altering [63–65]. Variants in vicinity of exon-intron junctions were therefore studied. After filtering (see Supporting Material), we found four variants of interest in the vicinity of splice sites (see S3 File). Two of these were in *MSH6* (NM_000179.2). The variant c.628-7C>A has been classified as VUS by us and ClinVar, The variant c.3439-1G>T, at the last nucleotide of 5^th^ intron, has been classified as pathogenic by ClinVar, it has been linked to LS/HNPCC phenotype, and has a maximum score (-1) for splice-site deactivation. We classified it as class 5 and hence disease causing.

An intronic variant in *MLH1* (NM_000249.3: c.306+4A>G) is found close to a splicing junction and was predicted for splice site deactivation. It is in a highly conserved position (PhastCons:1, phyloP:4.2), and has been classified as VUS in ClinVar. Experimental studies have shown that this variant results in the activation of a cryptic donor site and skipping of exon 3 in an ex-vivo splicing minigene assay [24], but as no studies have verified this in patient samples, we classified it as class 3 variant. An intronic variant in *MSH2* (NM_000251.2:c.1277-7C>A), previously classified as likely benign, we classified as a class 3 variant.

### UTR variants

There were 140 variants identified in UTR regions. Due to limitations of annotation tools and databases, any effects of most mutations in these regions are hard to predict. Hence, a relatively strict filtering compared to standard (for diagnostics) [66] was used for variants in these regions, to reduce the number of variants to a manageable size. After frequency-based filtering (< 0.01%) this list reduced to 28 variants, of which 9 variants were in 5’ and 19 variants were in 3’ UTR.

Variants in 5’ UTR were annotated for transcription factor binding sites (TFBSs), using the UniBind database [67]. Among the 9 variants in 5’ UTR, three had significant hits in the database, where each of these three variants was found inside a potential binding site for at least one transcription factor (TF) according to UniBind data (see S2 Table). One variant in *MSH6* (NM_000179.2: c.-76G>T) had overlap with potential binding sites for the TFs CTCF, STAT3, E2F7 and E2F1. For CTCF there is a high frequency of the reference allele (G) compared to the alternate allele (T) at the variant position, which can indicate a strong preference for the reference variant, and possibly a significant effect of the alternate variant on TFBS specificity (frequency matrices from the JASPAR database [68, 69] were used for this analysis). According to ChIP-seq data visualized with the UCSC genome browser [70] there are relatively strong signals for CTCF at this position (see S1 Fig) compared to other potential TFs. Mutations in CTCF binding sites have for example been associated with chromosomal instability and aberration and have been found in gastric and colorectal cancer [71], which strengthens the possibility that this variant may have an effect through altered binding of CTCF. A variant in *RFC3* (NM_002915.3: c.-106A>G) had hits for the TFs GABPA, JUN, CREM, JUND, ATF1, MITF, NR3C1, ATF7 and CREB1. Among these hits, 6 TFs (JUN, CREM, JUND, R3C1, ATF7 and CREB1) had a very high frequency of the reference allele (A) compared to the alternate allele (G) at the variant position. ChIP-seq data shows strong signals for CREB1 (see S2 Fig.), which may indicate a potential for significant effects due to alteration in the binding site. A variant in *RFC4* (NM_002916.3: c.-90C>T) had a hit for the TF AR.

Nineteen variants in 3’UTR were annotated using TargetScan v6.2 [72] and a two-step SVM prediction of micro-RNA (miRNA) target sites [73]. A SVM score normalization method [74] was used to normalize the score and miRNA data were taken from MirBase v22 [75]. Only a variant in gene *EXO1* (NM_130398.3:c.*84T>G) was predicted as a likely true candidate for affecting miRNA binding, for miR-370-3p and miR-93-3p (see S3 Table). Several studies have shown the importance of *EXO1* in replication, DNA repair pathways, cell cycle checkpoints and its association to cancer [76], and GWAS studies have identified specific mutations in *EXO1* gene as risk alleles for different types of cancer [77, 78]. SNPs in miRNA binding sites have been associated with CRC [79]. For the two miRNAs predicted to be affected by variation in their binding site, miR-370-3p has been identified as a tumour suppressor in EC via endoglin regulation [80]. The miR-93-3p can be considered as an important factor for CRC suppression and inhibition of tumorigenesis [81], as a previous study has associated the down-regulation of miR-93 with unfavourable clinicopathologic features and short overall survival of CRC patients [82].

### Implications of the study

In this study we found 35 significant variants (22 exonic, 4 UTR, 9 intronic), with 15 variants in the 4 MMR genes known to cause LS (*MLH1*, *MSH2*, *MSH6*, *PMS2*) and 20 in the additional MMR genes included in this study (*MSH3*, *POLD1*, *RFC1*, *RFC3*, *RFC4*, *LIG1*, *EXO1*, *RPA1*, *RPA3*). This helped in identification of variants in less studied genes, as well as polygenic variations (although none of the 199 samples in this particular study showed polygenic variants of interest for further investigations). This study also used the complete genomic regions of the genes, which very few previous studies have done [83, 84].

Though all known genes in the MMR pathway were studied, there will always be a possibility of additional genes and associated variants with similar disease effects, e.g., *POLE* mutations in EC cases contributing towards Polymerase Proofreading Associated Polyposis (PPAP) [85, 86], or germline deletions in another gene (*EPCAM1)* leading to silencing of the MSH2 gene, causing Lynch syndrome [87]. These limitations can only be removed by expanding the panel by including more genes, up to the extent of the whole genome. However, this will also increase the potential of noise and complexity of the analysis, by including more genes and variants that are less likely to be relevant in a given study. Another limitation is associated with the *PMS2* gene in this panel, which has a pseudo-gene (*PMS2CL*), and where 6 exons (exon 9, 11, 12, 13, 14 & 15) are highly similar to *PMS2CL*. This creates challenges in alignment of correct reads at these exons and creates artefacts during variant calling. This limitation has also been mentioned in a pilot study [83]. This makes it important to manually check reads and coverage in a genomic viewer, and to do Sanger verification of variants, as we did for the *PMS2* gene.

The current study emphasises the importance of including non-coding intronic regions. These regions will often have splice site variants, which may contribute to 10% of all disease-causing hereditary mutations according to HGMD [63–65], and deep intronic variants (e.g., in branch-point sequences, U2 type introns) which also contribute towards disease, most frequently by creating new pseudo-exons by activating non-trivial splice sites or by changing splicing regulatory elements. Intronic variants can also disrupt transcription regulatory motifs and non-coding RNA genes [88]. However, it is challenging to annotate these intronic variants due to limitations of annotation databases and tools. In a clinical setting, these variants can easily be missed unless RNA studies are performed to check for exon skipping, generation of new donor sites or cryptic site activation. Considering the potential importance of such variants, the current study included all intronic regions in order to search for this type of variant. Among 10 significant intronic variants we found four in the splice site vicinity and six in deep intronic regions.

NGS was performed, aiming at a data quality greater than 100X (average read coverage depth) for all samples. However, only 23 samples achieved this coverage (highest among them 169X), whereas 50 samples had coverage of less than 30X. These low-quality samples were included in the study, with the aim of exploring the value of low-quality data when searching for true positive (TP) variants. Using low quality data (i.e., with low coverage) led to a higher fraction of false positive (FP) variants, as 16 variants identified from the data analysis were subsequently identified as false positive variants by Sanger sequencing. Most of them had low coverage at the variant position (between 14X to 6X coverage), whereas others were in repeat regions. FP variants in *MLH1*, *MSH6* & *PMS2* genes were in repeat regions, and had low coverage (except a *PMS2* variant with 84X coverage), which possibly led to their false SNV call. On the other hand, we cannot rule out that some were not verified due to SNPs in the primer binding site (allelic dropout). However, we also found many true positive variants in low coverage regions, as we found and confirmed 6 true positive variants in regions with low coverage (between 16X and 4X). Among these were two class 3 variants (*MSH2*), one class 3+ variant (*POLD1*) and a class 5 variant (*MSH6*). This shows the potential for finding true variants of significance even in low-quality samples, given that the variants can be verified.

Our initial aim also included identification of CNVs. CNVs can occur in both exonic and intronic regions of protein coding genes, with intronic CNVs being more frequent [89], and both types can contribute towards disease. However, due to the limitations of data quality (non-uniform and low coverage depth), it was not possible to do reliable CNV calling. Also, there is no availability of MLPA kits (MRC-Holland) for detecting CNVs for many genes in this panel.

To associate variants with possible effects we utilized *in silico* resources and tools, in addition to published literature. Effect prediction and annotation of all variants was done using multiple tools as mentioned in the methods section. Also, multiple potential factors and effects, like conservation in variant position or structural changes at protein level, were checked for each variant. This consensus-like approach (multiple tools, multiple potential effects) increases the robustness of predictions and annotations of the variants, although we also had cases of contradictory predictions, which illustrates the challenge of using *in silico* prediction tools.

Among the 199 EC patient samples, we identified variants of interest (for further investigations) in 34 patients. Among these, we found 3 patients with class 5 variants (in *MSH6* gene) and two patients with the same class 4 variant (in *PMS2* gene); ~2,5% of patients had pathogenic variants representing a very likely cause of cancer in these five patients. This is in accordance with other studies. One meta-analysis of 53 studies concluded the prevalence of LS in EC patients to be approximately 3% [90]. These studies have only looked into the coding part of the four MMR genes MLH1, MSH2, MSH6 and PMS2. We found class 3 variants in another 29 patients, some of which are highly suspicious of being pathogenic variants. This indicates potential causes of their disease, although further studies are required to confirm their actual significance. It is an important limitation for further interpretation of these class 3 variants that we lack information about the patients debut age of cancer, and results from tumour analyses (MSI status and immunohistochemistry of MMR genes). For the remaining 164 patients, we did not find any significant variant to explain their disease. Expansion of panel size with more genes, improved annotation (particularly of variants in non-protein-coding regions), and improved data quality may help in explanation of some of these cases. However, since the study cohort consist of consecutive EC patients, most of the cancers will be sporadic with no underlying high penetrant genetic cause.

## Conclusions

Including all genes of the MMR pathway in a gene panel provides opportunity to discover variants in additional genes that potentially can be associated with a risk for EC, and hence are relevant for further investigation towards a better understanding of the development of EC. Including non-coding parts provides chances of identifying gene regulation or splice site alteration variants, although this will lead to a larger number of unknown variants which is challenging to study and annotate. In silico tools can be useful to find some leads in this situation, although their predictions can be ambiguous and noisy. Hence in silico tools should not be used in identifying pathogenicity by themselves. In addition, although low-quality data should be avoided, such data can still support identification of informative variants. But such data will also lead to increased noise in the analysis, and experimental verification of such variants is essential. We identified pathogenic MMR variants in the same order of magnitude as earlier reported. In addition, we identified 31 class-3 (VUS) variants some of which may be disease causing. This supports that screening for LS among EC patients should be recommended. However, to determine whether the use of an extended panel of MMR genes (beyond *MLH1*, *MSH2*, *MSH6* and *PMS2*) has clinical value needs further investigation.

## Supporting information

S1 Fig Transcription factor ChIP-seq cluster for MSH6 5’UTR variant. (TIF)

S2 Fig Transcription factor ChIP-seq cluster for RFC3 5’UTR variant. (TIF)

S1 Table Read coverage depth of samples across 12 sequencing runs. (PDF)

S2 Table List of 5’UTR variants. (PDF)

S3 Table List of 3’UTR variants. (PDF)

S1 File Bioinformatics analysis steps. (PDF)

S2 File Variants filtering steps. (PDF)

S3 File All significant variants with annotation details. (XLSX)
