# Supplementary figures and images for: Targeted sequencing of genes associated with the mismatch repair pathway in patients with endometrial cancer
Source: PLoS One. 2020 Jul 7;15(7):e0235613. doi: 10.1371/journal.pone.0235613 (PMC7340288; doi:10.1371/journal.pone.0235613)

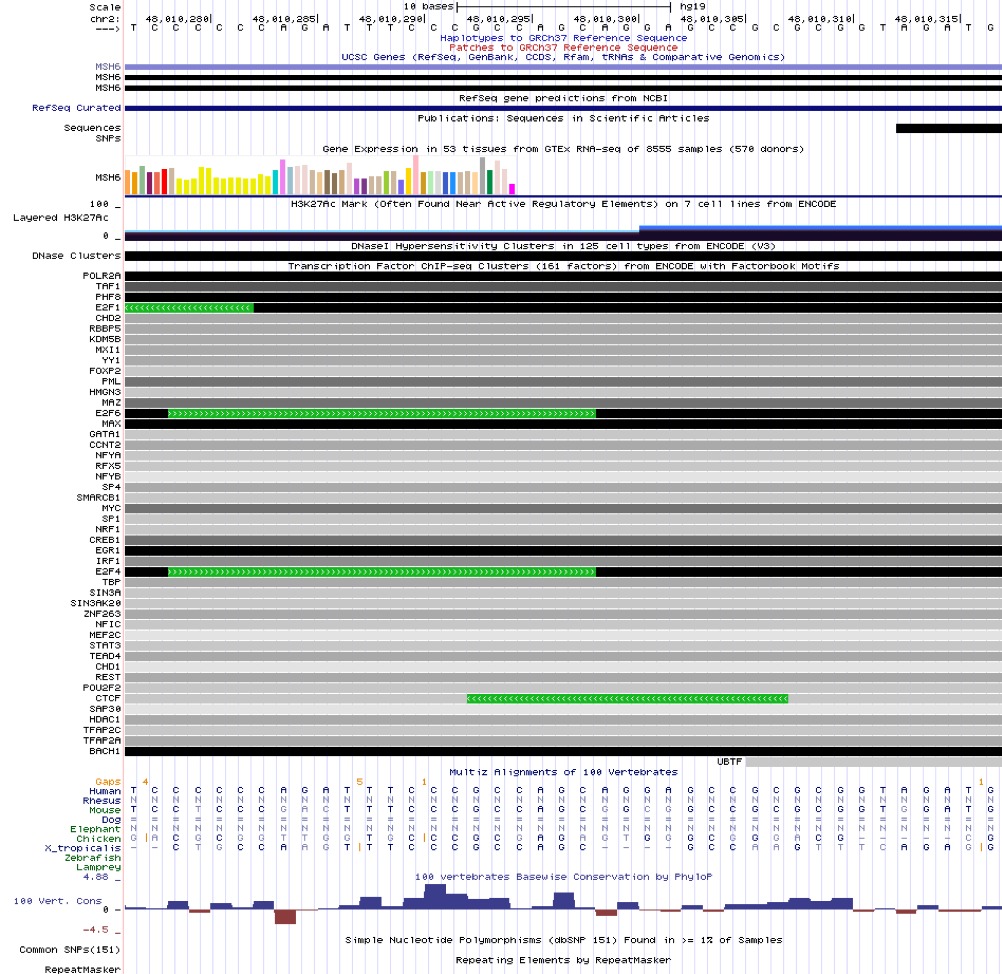

Supplement: S1 Fig — (TIF) [file pone.0235613.s001.tif]

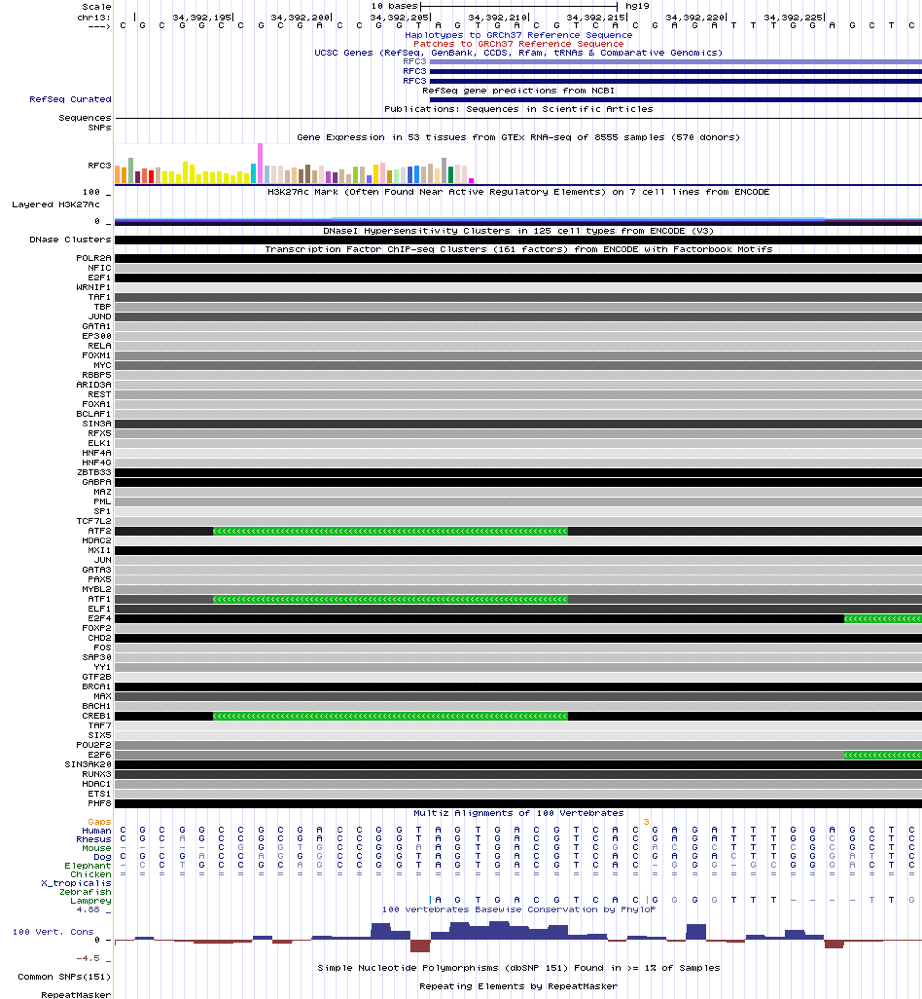

Supplement: S2 Fig — (TIF) [file pone.0235613.s002.tif]
