# Supplementary material for: Targeted sequencing of genes associated with the mismatch repair pathway in patients with endometrial cancer
Source: PLoS One. 2020 Jul 7;15(7):e0235613. doi: 10.1371/journal.pone.0235613 (PMC7340288; doi:10.1371/journal.pone.0235613)
Supplement: S1 Table — (PDF) [file pone.0235613.s003.pdf]

**Table S1:** Read coverage depth of samples across 12 runs

| Coverage Depth | Run1 | Run2 | Run3 | Run4 | Run5 | Run6 | Run7 | Run8 | Run9 | Run10 | Run11 | Run12 |
|---|---|---|---|---|---|---|---|---|---|---|---|---|
| **Mean coverage** (for run) | 79 | 89 | 74 | 11 | 10 | 30 | 28 | 43 | 71 | 51 | 96 | 38 |
| **Minimum coverage** (among samples) | 10 | 3 | 3 | 9 | 6 | 10 | 1 | 20 | 44 | 9 | 82 | 1 |
| **Maximum coverage** (among samples) | 169 | 126 | 124 | 27 | 20 | 42 | 65 | 79 | 11 | 130 | 128 | 75 |
