# Supplementary material for: Targeted sequencing of genes associated with the mismatch repair pathway in patients with endometrial cancer
Source: PLoS One. 2020 Jul 7;15(7):e0235613. doi: 10.1371/journal.pone.0235613 (PMC7340288; doi:10.1371/journal.pone.0235613)
Supplement: S2 Table — (PDF) [file pone.0235613.s004.pdf]

Table S2: 5'UTR variants

Variant	TF Name	TF Family	TF Class	JASPAR ID	TFBS Position (hg19)	TFBS Sequence	Strand	Count: A/ C/ G/ T	%A/ %C/ %G/ %T	Score: ref/alt	Score Change	Score%: ref/alt	Score% Change
**MSH6***<br>Chr2:48010297:G>T<br>NM_000179.2:c.-76G>T<br>PhastCons/phyloP:<br>0.984/2.304	CTCF	More than 3 adjacent zinc finger factors	C2H2 zinc finger factors	MA0139.1	Chr2:48010291-48010309	CCGCCA**G**CAGGAGCCGCGC	+	40/528/334/**11**	4.4/57.8/36.6/**1.2**	28.47/27.76	0.98	40.67/39.66	0.98
	STAT3	STAT factors	STAT domain factors	MA0144.2	Chr2:48010287-48010297	TTTCCCGCCA**G**	-	7312/3801/8571/**1936**	33.8/17.6/39.6/**9.0**	17.01/16.39	0.96	40.49/39.03	0.96
	E2F7	E2F-related factors	Fork head / winged helix factors	MA0758.1	Chr2:48010286-48010299	ATTTCCCGCCA**G**CA	+	930/1/3/**6**	98.9/0.1/0.3/**0.6**	22.95/22.96	1.00	44.14/44.15	1.00
	E2F1	E2F-related factors	Fork head / winged helix factors	MA0024.3	Chr2:48010287-48010298	TTTCCCGCCA**G**C	+	510/80/58/**182**	61.4/9.6/7.0/**21.9**	17.01/17.39	1.02	36.97/37.8	1.02
**RFC3**<br>chr13:34392210:A>G<br>NM_002915.3:c.-106A>G<br>PhastCons/phyloP:<br>1.000/2.707	GABPA	Ets-related factors	Tryptophan cluster factors	MA0062.2	Chr13:34392201-34392211	CCGGTAGTG**A**C	+	264/262/**415/**49	26.7/26.5/**41.9/**4.9	18.14/18.44	1.02	45.35/43.92	0.97
	JUN	Jun-related factors	Basic leucine zipper factors (bZIP)	MA0488.1	Chr13:34392204-34392216	GTAGTG**A**CGTCAC	+	20967/0/**0/**1	100.0/0.0/**0.0/**0.0	19.77/17.77	0.90	42.98/37.02	0.86
	CREM	CREB-related factors	Basic leucine zipper factors (bZIP)	MA0609.1	Chr13:34392206-34392215	AGTG**A**CGTCA	+	999/0/**0/**0	100.0/0.0/**0.0/**0.0	16.75/14.75	0.88	46.51/38.8	0.83
	JUND	Jun-related factors	Basic leucine zipper factors (bZIP)	MA0491.1	Chr13:34392206-34392216	AGTG**A**CGTCAC	+	38710/0/**0/**0	100.0/0.0/**0.0/**0.0	14.17/13.06	0.92	37.29/32.66	0.88
	ATF1	CREB-related factors	Basic leucine zipper factors (bZIP)	MA0604.1	Chr13:34392209-34392216	G**A**CGTCAC	-	712/0/**198/**90	71.2/0.0/**19.8/**9.0	14.06/13.03	0.93	50.21/43.43	0.86
	MITF	bHLH-ZIP factors	Basic helix-loop-helix factors (bHLH)	MA0620.2	Chr13:34392208-34392225	TG**A**CGTCACGAGATTTGG	-	9601/3103/**4895/**6354	40.1/13.0/**20.4/**26.5	26.59/26.2	0.99	41.55/39.7	0.96
	NR3C1	Steroid hormone receptors (NR3)	Nuclear receptors with C4 zinc fingers	MA0113.3	Chr13:34392206-34392222	AGTG**A**CGTCACGAGATT	+	18207/8/**9/**10	99.9/0.0/**0.0/**0.1	17.63/15.91	0.90	30.4/26.52	0.87
	ATF7	Jun-related factors	Basic leucine zipper factors (bZIP)	MA0834.1	Chr13:34392205-34392218	TAGTG**A**CGTCACGA	-	19329/77/**16/**25	99.4/0.4/**0.1/**0.1	22.24/20.25	0.91	44.47/38.94	0.88
	CREB1	CREB-related factors	Basic leucine zipper factors (bZIP)	MA0018.3	Chr13:34392206-34392217	AGTG**A**CGTCACG	+	8337/95/**292/**150	93.9/1.1/**3.3/**1.7	21.1/19.29	0.91	50.24/43.84	0.87
**RFC4**<br>chr3:186524157:G>A<br>NM_002916.3:c.-90C>T<br>PhastCons/phyloP: 0/1.013	AR	Steroid hormone receptors (NR3)	Nuclear receptors with C4 zinc fingers	MA0007.3	Chr3:186524145-186524161	AAGGACGAGATG**G**TCTC	-	**3/**3/5/2167	**0.1/**0.1/0.2/99.5	23.05/23.07	1.00	39.74/41.19	1.04
