# Supplementary material for: Targeted sequencing of genes associated with the mismatch repair pathway in patients with endometrial cancer
Source: PLoS One. 2020 Jul 7;15(7):e0235613. doi: 10.1371/journal.pone.0235613 (PMC7340288; doi:10.1371/journal.pone.0235613)
Supplement: S3 Table — (PDF) [file pone.0235613.s005.pdf]

Table S3: 3’UTR variants

GENE	SNPID	Transcript	MIRNA	DiffSc_SVM	Sc1_SVM	Sc2_SVM	DiffSc_TS	Sc1_TS	Sc2_TS	A1	SITE_A1	A2	SITE_A2	MiRExp	CANDIDATE
EXO1	chr1:242052986_T/G	NM_130398	miR-370-3p	0.9183	0	0.9183	0.477	0	-0.477	T	[ ]	G	[(CAGCA[G]GA 8m)]	medRpm	TRUE
			miR-93-3p	0.7675	0	0.7675	0.24	0	-0.24	T	[ ]	G	[(UCAGCA[G]G m8)]	medRpm	TRUE

*Table Description:

- SNPID: SNP ID with chromosome, position, ref allele, and alt allele
- DiffSc_SVM: Difference of SVM prediction scores between each allele: abs(Sc1_SVM - Sc2_SVM)
- Sc1_SVM: SVM prediction score of allele A1
- Sc2_SVM: SVM prediction score of allele A2
- DiffSc_TS: Difference of TargetScan prediction scores between each allele: abs(Sc1_TS - Sc2_TS)
- Sc1_TS: TargetScan prediction score of allele A1
- Sc2_TS: TargetScan prediction score of allele A2
- A1: Allele 1 (Ref Allele)
- SITE_A1: miRNA target site at allele A1
- A2: Allele 2 (Alt Allele)
- SITE_A2: miRNA target site at allele A2
- MiRExp: maximum miRNA expression level in FANTOM5 cell lines
- CANDIDATE: Possible filtering (MiRExp is highRpm or medRpm, MirSNP detected by both tools, and DiffSc_SVM>=0.4)

Target sites:

- - [] means no site at the allele
- - Each site is listed between parentheses: "(" and ")"
- - The 8mer sequence is the sequence of the 3'UTR mRNA which is reversed complement of the miRNA seed region (nucleotides 1-8 at the 5' end of the miRNA)
- - Within the sequence, the allele with the site is denoted within brackets (for instance [G])
- - After the space character, the seed type is denoted by:
- - "8m" for 8mer (sequence matching from miR nucleotides 1-8),
- - "m8" for 7merM8 (sequence matching from miR nucleotides 2-8)

Scores:

- - DiffSc_SVM and DiffSc_TS: the higher the better is the prediction of functional SNP
- - Sc1_SVM and Sc2_SVM: the higher the better is the prediction of miRNA target
- - Sc1_TS and Sc2_TS: the lower the better is the prediction of miRNA target
