# Supplementary material for: Targeted sequencing of genes associated with the mismatch repair pathway in patients with endometrial cancer
Source: PLoS One. 2020 Jul 7;15(7):e0235613. doi: 10.1371/journal.pone.0235613 (PMC7340288; doi:10.1371/journal.pone.0235613)
Supplement: S1 File — (PDF) [file pone.0235613.s006.pdf]

Bioinformatics analysis steps:
Data analysis analysis has 3 major components: Data preprocessing, Variant Discovery, Variants Refinement. Apart from data analysis, quality control was also done.

Work flow of complete analysis is as follows:

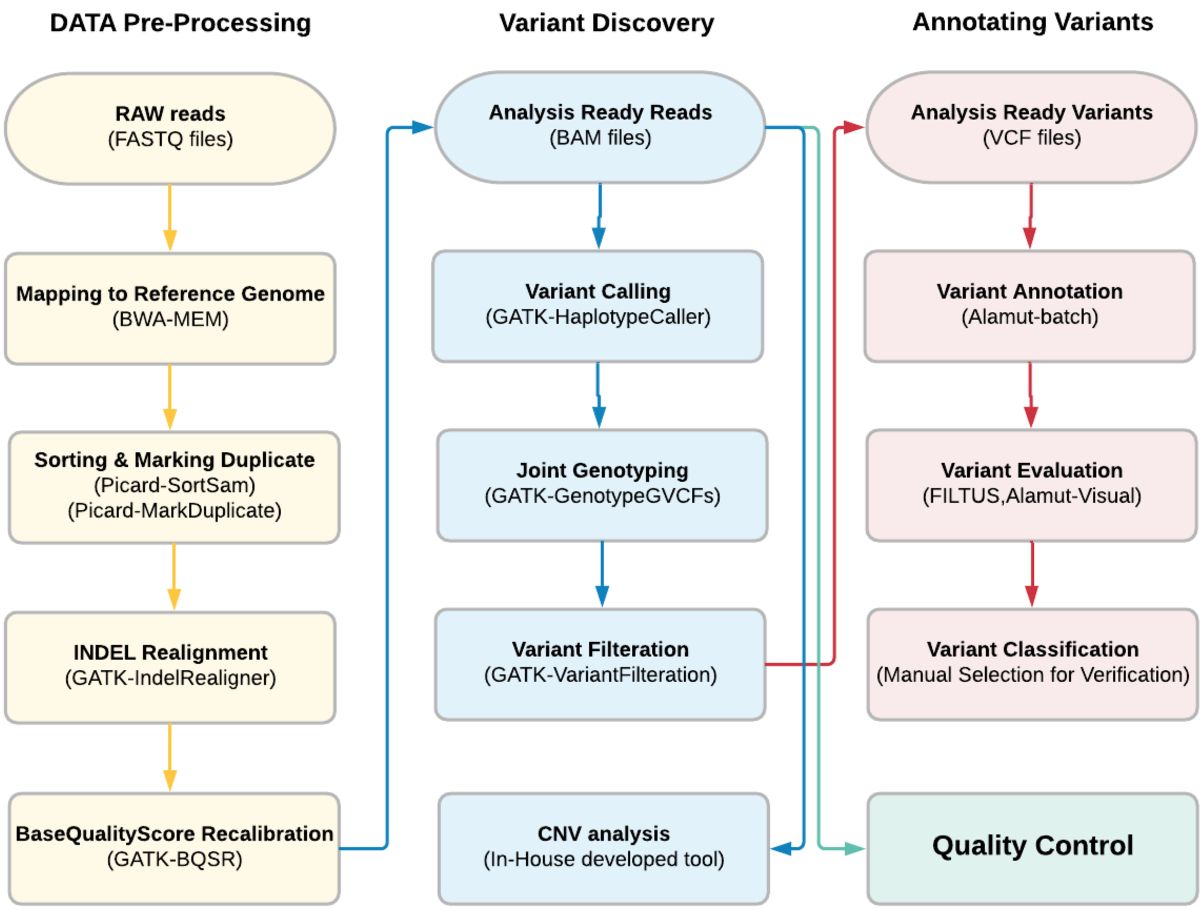

Details of tools, commands and options used for all 3 steps are as follows:

Data Preprocessing:

1. Mapping of RAW reads (as FASTQ files) to the reference genome (hg19 version)
   Tool used: BWA mem, Version=0.7.12-r1039
   Command:

```
bwa mem -M ucsc.hg19.fasta input_R1.fastq.gz input_R2.fastq.gz '>' aligned.sam
```

2. Sorting the aligned reads.
   Tool used: Picard-tools, Version=1.140
   Command:

```
java -jar picard.jar SortSam INPUT= aligned.sam OUTPUT= aligned_sorted.bam SORT_ORDER=coordinate
```

3. Marking the duplicates in BAM files.
   Tool used: Picard-tools, Version=1.140
   Command:

```
java -jar picard.jar MarkDuplicates INPUT= aligned_sorted.bam OUTPUT= aligned_sorted_dedup.bam METRICS_FILE=
 {}_metrics.txt
```

4. Adding header information to the BAM files
   Tool used: Picard-tools, Version=1.140
   Command:

```
java -jar picard.jar AddOrReplaceReadGroups I= aligned_sorted_dedup.bam O= aligned_sorted_dedup_RG.bam
SORT_ORDER=coordinate RGID= input_sample_name RGLB=bar RGPL=illumina RGPU=illumina_miSEQ RGSM= input_sample_name
CREATE_INDEX=True
```

5. Realignment of INDELs.

    a) Generating the targets which will be used for Indel realignment
       Tool used: GATK-RealignerTargetCreator,Version=3.4-46-gbc02625
       Command:

```
java -jar GenomeAnalysisTK.jar -T RealignerTargetCreator -R ucsc.hg19.fasta -I aligned_sorted_dedup_RG.bam -known
GATK_resources/Mills_and_1000G_gold_standard.indels.hg19.sites.vcf -known
GATK_resources/1000G_phase1.indels.hg19.sites.vcf -o INDEL_realigner.intervals
```

    b) Performing actual realignment
       Tool used: GATK-IndelRealigner,Version=3.4-46-gbc02625
       Command:

```
java -jar GenomeAnalysisTK.jar -T IndelRealigner -R ucsc.hg19.fasta -I aligned_sorted_dedup_RG.bam -known
GATK_resources/1000G_phase1.indels.hg19.sites.vcf -targetIntervals INDEL_realigner.intervals -o
aligned_sorted_dedup_RG_IndReAl.bam
```

6. Recalibration of base quality scores.

    a) Generating the recalibration table:
       Tool used: GATK-BaseRecalibrator,Version=3.4-46-gbc02625
       Command:

```
java -jar GenomeAnalysisTK.jar -T BaseRecalibrator -R ucsc.hg19.fasta -I aligned_sorted_dedup_RG_IndReAl.bam -knownSites
GATK_resources/dbsnp_138.hg19.vcf -knownSites GATK_resources/1000G_phase1.indels.hg19.sites.vcf -o base_recal.table
```

    b) Printing the recalibrated reads:
       Tool used: GATK-PrintReads,Version=3.4-46-gbc02625
       Command:

```
java -jar ~/my_tools/GATK/GenomeAnalysisTK.jar -T PrintReads -R ucsc.hg19.fasta -I aligned_sorted_dedup_RG_IndReAl.bam
-BQSR base_recal.table -o aligned_sorted_dedup_RG_IndReAl_Baserecal.bam
```

The final version of aligned BAM file is : "aligned_sorted_dedup_RG_IndReAl_Baserecal.bam" . It was used for further variants calling and visualization of reads

Variant discovery:
These steps were performed to generate the variant list.

1. Calling the variants by comparing the aligned reads to reference genome (hg19)
   Tool used: GATK-HaplotypeCaller,Version=3.4-46-gbc02625
   Command:

```
java -jar GenomeAnalysisTK.jar -T HaplotypeCaller -R ucsc.hg19.fasta -I aligned_sorted_dedup_RG_IndReAl_Baserecal.bam -o
raw_SNP_INDEL.g.vcf -mmq 0 -ERC GVCF --variant_index_type LINEAR --variant_index_parameter 128000 --dbsnp
GATK_resources/dbsnp_138.hg19.vcf -L targeted-regions.bed
```

   Note! Here targeted-regions.bed file consist of the regions that belong to 22 MMR-related genes
2. Genotyping the variants:
   Tool used: GATK-GenotypeGVCFs,Version=3.4-46-gbc02625
   Command:

```
java -jar GenomeAnalysisTK.jar -T GenotypeGVCFs -R ucsc.hg19.fasta -V raw_SNP_INDEL.g.vcf -o SNP_INDEL_genotyped.vcf
```

3. Quality Filter tagging (no variants were removed, only tagged).
   Tool used: GATK-VariantFilteration,Version=3.4-46-gbc02625
   Command:

```
java -jar GenomeAnalysisTK.jar -T VariantFiltration -R ucsc.hg19.fasta –variant SNP_INDEL_genotyped.vcf  -o
SNP_INDEL_genotyped_filtered.vcf --clusterWindowSize 10 --filterExpression "MQ0 >= 4 && ((MQ0/(1.0 * DP)) > 0.1)"
--filterName "HARD_TO_VALIDATE" --filterExpression "DP < 5 " --filterName "LowCoverage" --filterExpression "QUAL < 30.0 "
--filterName "VeryLowQual" --filterExpression "QUAL > 30.0 && QUAL < 50.0 " --filterName "LowQual" --filterExpression "QD
< 1.5 " --filterName "LowQD" --filterExpression "SB > -10.0 " --filterName "StrandBias"
```

The final file of variant calling step is : "SNP_INDEL_genotyped_filtered.vcf" . It was used for variants annotation.

Variant annotation:
This step was done to annotate the variants using Alamut annotation tool, which comprises on multiple annotation databases.

1. Annotating the variants with Alamut-batch.
   Tool used: Alamut-batch,Version=1.9
   Command:

```
alamut-batch-standalone-1.9/alamut-batch --in SNP_INDEL_genotyped_filtered.vcf --ann
SNP_INDEL_genotyped_filtered_annotated.vcf --unann SNP_INDEL_genotyped_filtered_unannotated.vcf --donnsplice
--dogenesplicer --ignoreInputErrors
```

The final annotated variant file is "SNP_INDEL_genotyped_filtered_annotated.vcf". It was used for the classification steps.

Quality Control:

These steps were done to generate the quality statistics of the sequencing data, aligned reads, & variants. Which are as follows.

1. Running the GATK-DepthOfCoverage command to generate coverage statistics of reads.
   Tool used: GATK-DepthOfCoverage,Version=3.4-46-gbc02625
   Command:

```
java GenomeAnalysisTK.jar -T DepthOfCoverage -R ucsc.hg19.fasta -I aligned_sorted_dedup_RG_IndReAl_Baserecal.bam -o
SAMPLE_ID -L targeted-regions.bed
```

   This will generate 7 quality control files, which are:
   - SAMPLE_ID
   - SAMPLE_ID.sample_summary
   - SAMPLE_ID.sample_interval_summary
   - SAMPLE_ID.sample_cumulative_coverage_counts
   - SAMPLE_ID.sample_cumulative_coverage_proportions
   - SAMPLE_ID.sample_interval_statistics
   - Sample_ID.sample_statistics
2. Running Samtools-flagstat to generate the mapping statistics
   Tool used: samtools-flagstat,Version=1.2
   Command:

```
samtools flagstat aligned_sorted_dedup_RG_IndReAl_Baserecal.bam > SAMPLE_ID_mapping_statistics.txt
```

   This step generate the reads statistics for aligned/unaligned (to reference genome hg19) reads.

File Format details:

- FASTQ (.fastq): are the file format for short read sequences coming out of sequencer.
- SAM (.sam): Sequence Alignment Maps, are aligned reads to the reference genome
- BAM (.bam): Binary Alignment Maps are binary version of SAM files
- VCF (.vcf): Variant calling Format, are for the variants files.
- BED (.bed): are tab-delimited text files that defines a feature track for the target regions.

List of used Tools:

- BWA [1]
- Picard Tools [2]
- Samtools [3]
- GATK toolkit [4]
- Alamut-batch [5]

References

1. Li H, Durbin R. Fast and accurate short read alignment with Burrows-Wheeler transform. Bioinformatics. Oxford University Press; 2009;25: 1754–1760. doi:10.1093/bioinformatics/btp324

2. Broad Institite. Picard Tools. Available: http://broadinstitute.github.io/picard/

3. Li H, Handsaker B, Wysoker A, Fennell T, Ruan J, Homer N, et al. The Sequence Alignment/Map format and SAMtools. Bioinformatics. Oxford University Press; 2009;25: 2078–2079. doi:10.1093/bioinformatics/btp352

4. McKenna A, Hanna M, Banks E, Sivachenko A, Cibulskis K, Kernytsky A, et al. The Genome Analysis Toolkit: a MapReduce framework for analyzing next-generation DNA sequencing data. Genome Res. Cold Spring Harbor Laboratory Press; 2010;20: 1297–303. doi:10.1101/gr.107524.110

5. Alamut. Alamut-batch [Internet]. Alamut; Available: https://www.interactive-biosoftware.com/alamut-batch/
