# Supplementary material for: Targeted sequencing of genes associated with the mismatch repair pathway in patients with endometrial cancer
Source: PLoS One. 2020 Jul 7;15(7):e0235613. doi: 10.1371/journal.pone.0235613 (PMC7340288; doi:10.1371/journal.pone.0235613)
Supplement: S2 File — (PDF) [file pone.0235613.s007.pdf]

Variant filtering steps:

Total 199 unique samples were used in study. Total 10680 unique variants were found after variant-calling step.

All variants were annotated using Alamut-batch software [1] and filtering was done based on these annotations. Filtus software [2] was used to do the filtering.

Filtering work-flow:

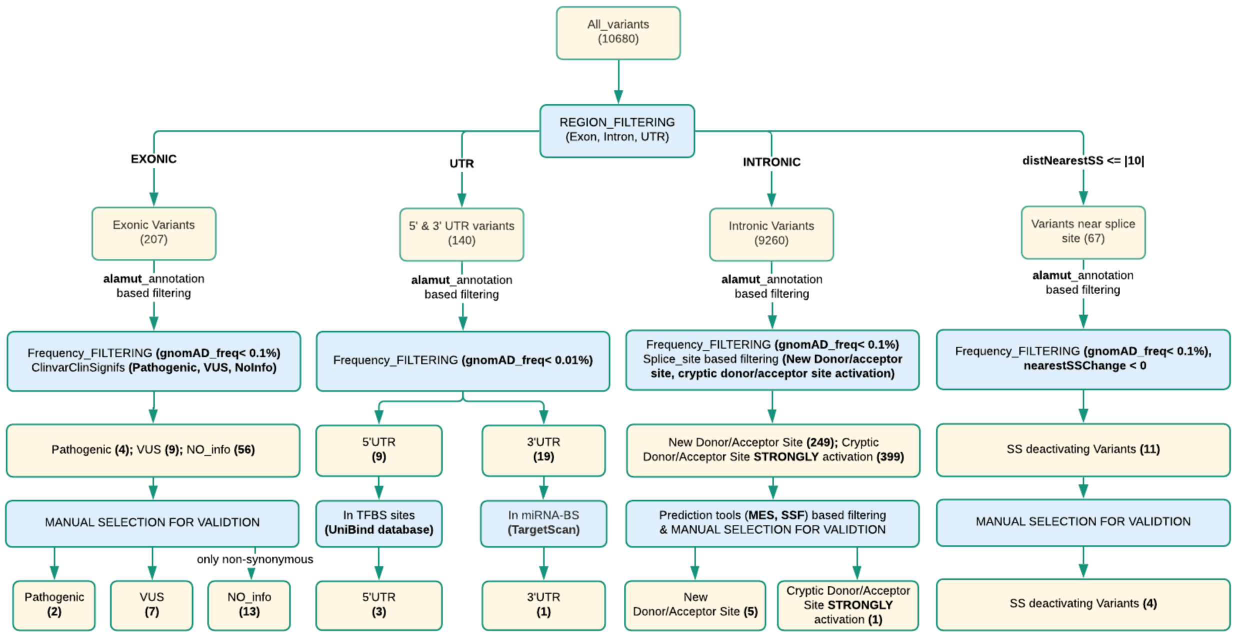

- **Exonic Variants:**
  - Exonic variants: filtering criteria: *"varLocation equal to exon"*
  - Frequency based filtering: filtering criteria: *"gnomadAltFreq_all less then 0.001"*
  - Clinvar [3] based filtering: filtering criteria: *"ClinVarClinSignifs does not contain benign", "ClinVarClinSignifs does not contain Benign"*

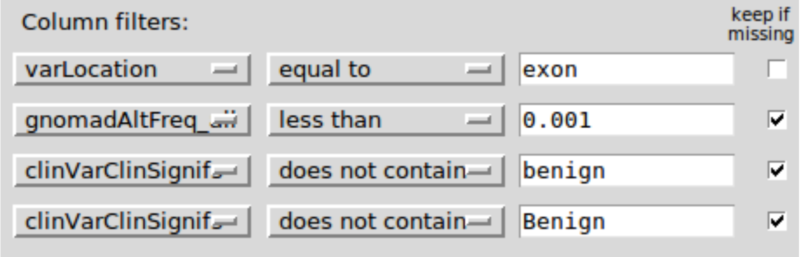

Further final results of filtering was classified in three categories (as per ClinVar classification)

- Pathogenic variants
- VUS (variants of unknown significance) variants
- No_info (No Information) variants

- **UTR region Variants:**
  - UTR regions variants: filtering criteria: *"varLocation contains UTR"*
  - Frequency (from gnomAD [4])based filtering: filtering criteria: *"gnomadAltFreq_all less then 0.0001"*

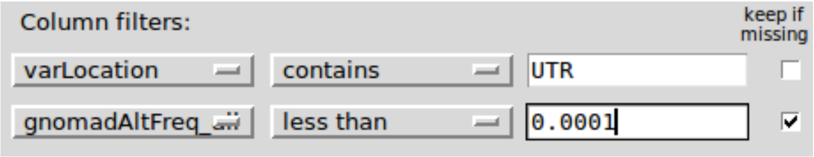

Further final results of filtering was classified in 5’UTR & 3’UTR regions based variants.

- **Intronic Variants:**
  - Intronic Variants: filtering criteria: *“varLocation equal to intron”*
  - Frequency based filtering: filtering criteria: *“gnomadAltFreq_all less then 0.001”*
  - Splice Site based Filtering:
    - New Donor/Acceptor Site: filtering criteria: “localSpliceEffect contains New”
      - splice site prediction tools (Splice Site Finder [5] & MaxEntScan[6]) based filtering: filtering criteria: “Local_Var_MES_score greater then 8”, “Local_Var_SSF_score greater then 80”.

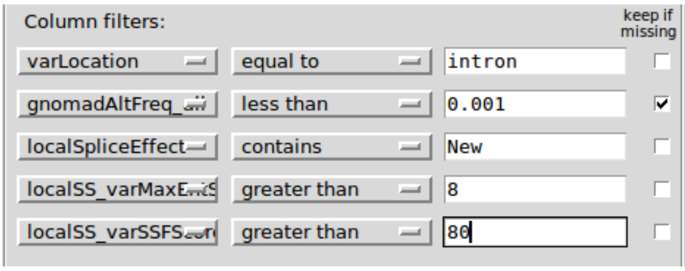

    - Cryptic Donor/Acceptor site activated: filtering criteria: “localSpliceEffect contains Strongly”
      - splice site prediction tools based filtering: *filtering criteria: “Local_Var_MES_score greater then 8”, “Local_Var_SSF_score greater then 80”, “ Diff%_Local_WT-vs-Var_MES_score less then -20%”, Diff%_Local_WT-vs-Var_SSF_score less then -4%* ”. (here % difference was calculated manually and added to the variant files)

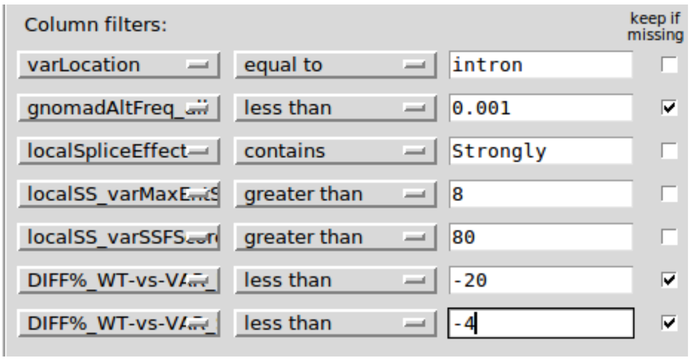

- **Splice site region Variants (distNearestSS ≤ |10|):**
  - Splice site region Variants: filtering criteria: *“distNearestSS less then 11”, “distNearestSS greater then -11)”*
  - Frequency based filtering: *filtering criteria: “gnomadAltFreq_all less then 0.001”*
  - Splice Site based Filtering (DE-ACTIVATING variants): filtering criteria: “nearestSSChange less then 0”

References:

1. Alamut. Alamut-batch [Internet]. Alamut; Available: https://www.interactive-biosoftware.com/alamut-batch/

2. Vigeland MD, Gjøtterud KS, Selmer KK. Data and text mining FILTUS: a desktop GUI for fast and efficient detection of disease-causing variants, including a novel autozygosity detector. doi:10.1093/bioinformatics/btw046

3. Landrum MJ, Lee JM, Riley GR, Jang W, Rubinstein WS, Church DM, et al. ClinVar: public archive of relationships among sequence variation and human phenotype. doi:10.1093/nar/gkt1113

4. Lek M, Karczewski KJ, Minikel E V., Samocha KE, Banks E, Fennell T, et al. Analysis of protein-coding genetic variation in 60,706 humans. Nature. Nature Publishing Group; 2016;536: 285–291. doi:10.1038/nature19057

5. Shapiro MB, Senapathy P. RNA splice junctions of different classes of eukaryotes: sequence statistics and functional implications in gene expression. Nucleic Acids Res. Oxford University Press; 1987;15: 7155–74. Available: http://www.ncbi.nlm.nih.gov/pubmed/3658675

6. Yeo G, Burge CB. Maximum Entropy Modeling of Short Sequence Motifs with Applications to RNA Splicing Signals. J Comput Biol. 2004;11: 377–394. doi:10.1089/1066527041410418
